# Teachers' Understanding of the Role of Executive Functions in Mathematics Learning

**DOI:** 10.1111/mbe.12050

**Published:** 2014-08-18

**Authors:** Camilla Gilmore, Lucy Cragg

**Affiliations:** 1 Mathematics Education Centre, Loughborough University; 2 School of Psychology, University of Nottingham

## Abstract

Cognitive psychology research has suggested an important role for executive functions, the set of skills that monitor and control thought and action, in learning mathematics. However, there is currently little evidence about whether teachers are aware of the importance of these skills and, if so, how they come by this information. We conducted an online survey of teachers' views on the importance of a range of skills for mathematics learning. Teachers rated executive function skills, and in particular inhibition and shifting, to be important for mathematics. The value placed on executive function skills increased with increasing teaching experience. Most teachers reported that they were aware of these skills, although few knew the term “executive functions.” This awareness had come about through their teaching experience rather than from formal instruction. Researchers and teacher educators could do more to highlight the importance of these skills to trainee or new teachers.

One challenge for cognitive psychology research is to reveal the processes and mechanisms that are important for successful learning. In the past two decades the learning of mathematics, in particular, has received increasing attention. Psychologists have identified a number of skills that are related to successful mathematics achievement. These include both domain-specific skills such as knowledge of number facts and conceptual understanding (e.g., Cowan et al., 2011) as well as domain-general abilities (e.g., Fuchs et al., 2010; LeFevre et al., 2010).

Particular attention has been paid to executive function skills, the set of skills that monitor and control thought and action (see reviews by Cragg & Gilmore, 2014; Friso-van den Bos, van der Ven, Kroesbergen, & van Luit, 2013; Raghubar, Barnes, & Hecht, 2010). These skills include monitoring and manipulating information in mind (working memory), suppressing distracting information and unwanted responses (inhibition), and flexible thinking (shifting). These skills are related to performance on mathematics achievement tests and change in mathematical performance over time.

Evidence for the role of executive function skills comes from studies using cognitive, experimental tasks to measure participants' executive function skill. Scores from these tests are related to concurrent or future mathematics achievement. In the majority of cases, participants take part in studies in controlled situations away from the classroom. Therefore, these studies do not show whether the role of executive functions is also evident in everyday classroom situations. To date there is no evidence whether teachers are aware of the importance of executive function skills in mathematics learning and, if so, how they come by this information. Teachers' classroom experience may play a role in this and thus we would expect more experienced teachers to be more aware of these skills. It is also possible that these skills are evident to a different extent depending on the age group of the children being taught.

Exploring teachers' understanding of the role of executive functions in mathematics is important for two reasons. First, evidence has demonstrated that teachers' conceptions of child development and their knowledge of neuroscience influence the teaching practices they employ (Daniels & Shumow, 2003; Dubinsky, Roehrig, & Varma, 2013). Therefore, it is plausible that teachers who are aware of the importance of executive function skills may modify their behavior to reduce the executive function demands placed on their students. Second, in order for research evidence to have an impact on educational practice, researchers need to understand teachers' conceptions of these issues. Presently little is known about this in regard to cognitive psychology, although research has demonstrated that teachers' knowledge of educational neuroscience is generally poor and teachers are likely to hold many misconceptions (so-called *neuromyths*; Dekker, Lee, Howard-Jones, & Jolles, 2012).

We therefore carried out a survey to discover UK teachers' understanding of executive functions, with two main aims. First, we explored what skills teachers consider to be important for learning mathematics. Second, we ascertained whether teachers were aware of research evidence for the role of executive functions in mathematics and how they had developed this awareness.

## Method

### Participants

We conducted an online survey of teachers' knowledge of executive functions. The survey was active for 1 month and advertised on UK teacher websites and forums and sent to personal and professional contacts of the authors. A total of 96 teachers completed the survey. The survey was open to teachers from all school stages: 8% taught foundation stage (age up to 5), 52% taught in primary or junior schools (age 6–11), and 40% taught in secondary schools (age 11–18). Teachers who completed the survey also varied in years of teaching experience: 1% were in training, 29% had 1 to 3 years experience, 35% had 4 to 10 years experience, and 34% had more than 10 years experience. Just over half of respondents (54%) taught mathematics as their main subject or specialism. In the UK, all foundation and primary school teachers are required to teach mathematics, but in secondary school mathematics is only taught by specialists. Our sample had a higher proportion of primary school teachers (52% vs. 33%), and within secondary schools, a higher proportion of mathematics specialist teachers (87% vs. 14%) compared to the national population (Department for Education, 2012).

### Materials

Teachers completed a Mathematics Skills Questionnaire in which they rated the importance of 12 basic skills for mathematics learning on a 5-point Likert scale from 0 (*not important*) to 4 (*extremely important*). Four items referred to mathematics-specific (MS) skills, four items referred to executive function (EF) skills, and four items referred to other skills (OS) (Table 1). The different types of items were intermixed. Subsequently teachers were asked whether they had heard of the term *executive functions*, if they were previously aware of the importance of these types of skills for learning mathematics and, if so, how they had learned this (e.g., through training or experience).

**Table 1:** Items in the Mathematics Skills Questionnaire With Mean Rating (Maximum 4) and Factor Loadings.

*Domain*	*Items*	*Mean rating*	*Factor loading*
Mathematics-specific (MS)	Know number facts	3.29	.76
	Understand mathematical concepts	3.38	.61
	Understand how mathematics is used in the real world	3.00	.70
	Know formulas and procedures	2.56	.55
Executive functions (EF)	Manipulate abstract information	2.64	.76
	Store and manipulate information in their head	2.68	.74
	Focus on relevant information and avoid distractions	3.05	.67
	Be able to think flexibly	3.02	.80
Other skills (OS)	Have good verbal skills	2.53	.86
	Be able to provide reasons to support their solutions	3.22	.82
	Be able to think creatively	2.76	.78
	Have good spatial skills	2.48	.75

Note. Teachers Were Asked: “To be good at mathematics at school, how important do you think it is for students to…”

The questionnaire items were selected by drawing on research evidence. The EF items referred to working memory, inhibition, and shifting (Huizinga, Dolan, & van der Molen, 2006; Miyake et al., 2000). The MS items referred to conceptual, procedural, and factual knowledge (Dowker, 2005) and the distinction between abstract and concrete mathematics skills (Kaminski, Sloutsky, & Heckler, 2008). The OS items included skills that are not specific to mathematics but are frequently associated with mathematics achievement, either in research evidence or more informally, including verbal and spatial abilities and creativity.

## Results

### Mathematics Skills Questionnaire Construction

Mean importance ratings for the 12 items are given in Table 1. Initially we explored whether the items in each domain (MS, EF, and OS) measured the same construct. We conducted a principal components analysis for each of the domains to explore whether all items for that domain loaded strongly onto a single factor and variable loadings are given in Table 1. For each of the domains a single factor was extracted that accounted for substantial variance (MS items 44%, EF items 55%, OS items 65%). Internal consistency was good for the EF and OS items (α = .73 and α = .81, respectively) but lower for the MS items (α = .56), which may reflect the multicomponential nature of mathematics ability. Mean scores for each domain were used in the analyses, although the same results were obtained using factor scores.

### Skill Importance Ratings

Teachers' responses were explored with a series of analysis of variances (ANOVAs) and follow-up Bonferroni-corrected *t*-tests. A one-way ANOVA revealed that teachers' mean importance ratings for the three domains differed, *F*(2, 180) = 12.9, *p* < .001. MS items (*M* = 3.05) were rated as more important than EF items (*M* = 2.85, *p* = .003), or OS items (*M* = 2.74, *p* < .001), which did not differ (*p* = .128).

We then explored whether participant characteristics (teaching experience, school type) affected responses for each domain. A two-way ANOVA was conducted with domain (MS, EF, and OS) and years of teaching experience (1–3, 4–10, >10) as factors. There were significant effects of teaching experience, *F*(2, 87) = 3.69, *p* = .029, and domain, *F*(2, 174) = 13.35, *p* < .001, and the interaction approached significance, *F*(4, 174) = 2.08, *p* = .086. Teaching experience had a significant effect on importance ratings for the MS items, *F*(2, 89) = 4.62, *p* = .012, and EF items, *F*(2, 91) = 5.92, *p* = .004, but not OS items, *F*(2, 89) = 2.52, *p* = .09. For MS skills, teachers with 1 to 3 years experience gave lower importance ratings (*M* = 2.77) than teachers with either 4 to 10 (*M* = 3.17, *p* = .029) or more than 10 years experience (*M* = 3.19, *p* = .023), who did not differ (*p* = .99). In contrast, for EF skills, only teachers with more than 10 years experience (*M* = 3.10) gave higher ratings than those with less than 3 years experience (*M* = 2.52, *p* = .003), whereas ratings from teachers with 4 to 10 years experience (*M* = 2.86) did not differ from either less (*p* = .133) or more experienced (*p* = .412) teachers.

A second two-way ANOVA was conducted with domain (MS, EF, and OS) and school type (foundation, primary, secondary) as factors.^1^ There were significant effects of domain, *F*(2, 176) = 16.30, *p* < .001, and school type, *F*(2, 88) = 4.91, *p* = .009, which were modified by a significant interaction, *F*(4, 176) = 2.59, *p* = .039. School type did not significantly impact importance ratings for EF items, *F*(2, 90) = 2.82, *p* = .065, or OS items, *F*(2, 90) = 1.51, *p* = .227, but did affect ratings for MS items, *F*(2, 90) = 10.32, *p* < .001. Teachers in secondary schools rated MS skills as less important (*M* = 2.76) than teachers in primary schools (*M* = 3.20, *p* = .001) or foundation stage (*M* = 3.56, *p* = .001), whose ratings did not differ (*p* = .276). This is likely to reflect differences in the nature of mathematics content across these age groups.

We next explored ratings within the EF domain to discover which individual skills were considered most important and whether this differed for teachers with different levels of experience (see Table 1 for mean ratings). We conducted a two-way ANOVA with skill (inhibition, shifting, working memory storage, and working memory processing) and years of teaching experience (1–3, 4–10, >10) as factors. There were significant effects of experience, *F*(2, 91) = 5.92, *p* = .004, and skill, *F*(3, 273) = 9.28, *p* < .001. Teachers rated inhibition skills (“Focus on relevant information and avoid distractions”) as more important than working memory skills (“Manipulate abstract information,” *p* = .002, “Store and manipulate information in their head,” *p* = .006). They also rated shifting skills (“Be able to think flexibly”) as more important than both working memory items (*p* = .001 and *p* = .009 respectively). They did not differentiate between inhibition and shifting skills (*p* = .999) or between the working memory items (*p* = .999). There was no interaction between skill and teaching experience, *F*(6, 273) < 1.

### Awareness of Executive Functions

Only 18% of teachers reported that they had heard of the term *executive functions* before completing the survey; however, 72% indicated they had some awareness that these types of skills were important. Level of awareness increased with teaching experience (χ^2^ = 7.42, *p* = .025). Teachers who had more years of teaching experience were more likely to report that they knew *a little* or *a lot* about the role that executive function skills play. In keeping with this, the majority of teachers reported that they had learnt this from their own teaching experience (63%), rather than during their initial teacher training or later professional development (16%).

## Discussion

Our findings give a picture of UK teachers' knowledge and understanding of the role of executive function skills in mathematics learning. Teachers rated all of the skills in our survey as somewhat important, but differentiated between the specific skills that we explored. It is clear that, even without formal instruction in this topic, the majority of teachers are aware that skills such as holding and manipulating information in mind (working memory), ignoring distractions (inhibition), and thinking flexibly (shifting) are important, even if they have never heard the term “executive functions.” This demonstrates that the importance of these skills is apparent in the classroom as well as in the laboratory. Our findings are based on a small-scale convenience sample and it would be helpful to replicate these effects with a larger sample that is fully representative of the population of teachers.

Within the domain of executive functions, it is interesting to note that teachers identified inhibition and shifting skills as being more important than working memory. This is based on responses to individual items and thus this pattern should be validated in further research with more extensive questionnaires. However, it is interesting to note that this pattern is somewhat in contrast to research evidence, which has tended to focus on the role of working memory in mathematics learning (Raghubar et al., 2010). However, recent research is beginning to explore the important roles of inhibition (Gilmore et al., 2013) and switching skills (e.g., Yeniad, Malda, Mesman, van IJzendoorn, & Pieper, 2013). Our findings suggest that more research is needed to understand better how these skills help with learning mathematics. One reason for the contrast between research findings and teachers' views may be the nature of the tasks used. Researchers tend to focus on abstract cognitive tasks, which may not tap into how children's skills manifest in the classroom. Recent research has begun to develop more real-life executive tasks (e.g., Yang, Gathercole, & Allen, 2013) and these may help shed light on the way that executive functions support mathematics learning.

Our findings show that it may take many years of experience for teachers to become aware of the importance of executive function skills. While teachers' ratings of the importance of different mathematics-specific skills increased with relatively few years of teaching experience (>4), ratings of the importance of executive function skills only increased after more than 10 years of teaching experience. Given these results, it is important to consider whether teacher education courses provide adequate training in understanding the importance of these skills.

Teachers in our survey who were aware of the importance of executive function skills reported that they had learnt this through their own teaching experience and not during their training. This is in keeping with research showing that student teachers fail to learn about child development during their training (McDevitt & Ormrod, 2008). Laski, Reeves, Ganley, and Mitchell (2013) recently surveyed teacher educators about the cognitive psychology content of their courses, and their attitudes to cognitive research more generally. They found that although teacher educators view cognitive studies of mathematics to be fairly important for mathematics education, they placed less emphasis on cognitive psychology research more generally. This suggests that topics such as executive functions may not appear in mathematics teacher training.

Why might cognitive psychology research be undervalued in teacher education? Laski et al. (2013) found that the extent to which teacher educators engaged with cognitive research and included it in teacher training courses was related to their perceptions of the importance of this research. It is, therefore, vital that cognitive psychology researchers demonstrate to both teachers and teacher educators how and why research on the role of cognitive processes such as executive function is important for mathematics learning and how educational practices can build on this knowledge. Our findings suggest that it would be beneficial for teachers to be given this information during training or early in their teaching career as it can take 10 years for teachers to discover it from classroom experience. To maximize the classroom impact of research in this area, researchers need to be aware of the context their research impacts upon and use this information when talking with educators. Having a clearer picture of teachers' understanding of topics such as cognitive psychology will help to achieve this.

## Note

1 Given the structure of the UK education system, mathematics specialists and nonspecialists were not evenly distributed across school types. Therefore, it is not possible to disentangle the effects of these factors.
